# An Intelligent Joint Identification Method and Calculation of Joint Attitudes in Underground Mines Based on Smartphone Image Acquisition

**DOI:** 10.3390/s25206410

**Published:** 2025-10-17

**Authors:** Guang Li, Jinyao Zhu, Changyu Jin, Xinyang Mao, Qiang Wang

**Affiliations:** State Key Laboratory of Intelligent Deep Metal Mining and Equipment, Northeastern University, Shenyang 110819, China; liguang_angang@163.com (G.L.); jinchangyu@mail.neu.edu.cn (C.J.); maoxinyang6@gmail.com (X.M.); wangqiangzitu@mail.neu.edu.cn (Q.W.)

**Keywords:** geologic description, joint identification, deep learning, image segmentation, attitude calculation

## Abstract

Acquisition of joint attitudes is vital in mine geology but often constrained by underground conditions, while manual cataloging remains inefficient and subjective. To overcome these issues, we propose a mobile phone photography and deep learning-based method. Rock joint images are collected with smartphones, augmented by cutting and rotation, and enhanced using CLAHE. After labeling with Labelme, a dataset is built for training. A ResNet residual module and CBAM attention are integrated into a U-Net architecture, forming the RC-Unet model for accurate semantic segmentation of joints. Post-processing with OpenCV enables contour extraction, and the PCP three-point localization algorithm rapidly calculates joint attitudes. A practical engineering case verifies that intelligent joint identification can replace manual cataloging in relatively simple underground environments. This approach improves efficiency, reduces subjectivity, and provides a rapid, low-cost, and easily storable means for geological information acquisition, highlighting its potential as an effective tool and supplementary method for mine surveys.

## 1. Introduction

Joint identification and attitude calculation of rocks are important tasks in geological cataloging of mines, with the purpose of supplementing the geological survey in the exploration stage [1]. The accuracy of joint description in rocks not only directly affects the quality of geological prospecting and the progress of geological research, but also is closely related to mine construction and mining safety. The conventional geological cataloging methods mainly depend on artificial measurement, which is not only inefficient but also may cause a security threat [2]. With the development of the cataloging technique, the geological cataloging based on photography has become the main direction of research. The typical methods include the geological cataloging based on ordinary cameras, which uses ordinary cameras to take engineering images, supplemented by artificial cataloging; geological cataloging based on close-range photogrammetry, which uses a plotting apparatus and a coordinatograph to establish a three-dimensional (3D) measurement system, to realize spatial positioning and image acquisition of cataloged information. However, when applied to practical engineering, the two methods face two problems: low operability and high cost of photographic equipment, and heavy dependence of image information processing on artificial work. At present, there is still a lack of effective, smart image processing methods that can realize targeted and efficient extraction of information for geological cataloging.

With the rapid development of smartphone hardware, underground imaging has become increasingly convenient and can take high-definition photos of joints on rock walls even in poorly lit environments; additionally, deep learning provides a new idea and method for geological cataloging due to its strong learning ability and feature extraction capability. Therefore, recognizing geological information of rocks and structural planes in images on the basis of deep learning [3] has become a new development direction of geological cataloging of mines.

At present, numerous researchers have used image segmentation models based on deep learning [4] (such as fully convolutional network (FCN) [5] and U-shaped network (U-Net) [6] ) to identify the attitude of joints and fractures under various scenarios and achieved fruitful results. Xue et al. recognized traces on rock surfaces using FCN and verified that FCN is able to efficiently identify most fractures on rock surfaces. By employing the convolutional neural network (CNN), Xue and Li [7] and Huang and Li [8] identified fractures on roadway lining, and the identification accuracy exceeded 80%. Ref. [9] adopted the conventional U-Net to decode and encode fractures on the bituminous pavement and obtained favorable identification results, solving the problem of rough fracture edges on slopes. Based on the attention mechanism, Hanat and Liu [10] improved the FCN, and the improved FCN shows a better identification effect on complex fractures on the concrete surface. Zhang [11] improved the conventional U-Net through dilated convolution and extracted geometrical parameters of fractures based on GMM-EE and RANSAC algorithms. In this way, they achieved the rapid acquisition of fracture information on the surface of high and steep rock slopes. Karimpoul and Tahmasebi [12] proposed the convolutional autoencoder to improve the segmentation effect on digital rock images and increased the necessary dataset via simulation based on cross-correlation. The classification accuracy of the extended network for rock images reached 96%, which was qualitatively compared with the results of conventional multiphase segmentation (multi-threshold segmentation), verifying the better segmentation results of the proposed method. Song [13] came up with the improved U-Net deep convolutional network, which was used to segment gray-scale electron microscope (EM) images and seabed mineral images. The accuracy of the network for the EM image dataset reached 91.6%, and its segmentation effect on the seabed mineral image dataset is superior to the original U-Net convolutional network. Limited by the underground lighting condition and shooting angle, joint images are generally characterized by a small gray-scale difference, complex joint shape, and unclear images, so that the existing deep learning method still remains to be improved in terms of the joint identification accuracy and precision in such images. Therefore, it is necessary to establish a deep learning model that couples the geometrical features of joints in a bid to rapidly and accurately recognize joints in the images.

Moreover, in the research on attitude calculation methods of 3D structural planes, many researchers have used different methods to gain many results. Taking a rectangular cavern as an example, Chen [14] described the concrete implementation processes of measurement based on the stereopair technique and orthophotography. Lin [15] found that the geographic coordinates and elevation information of arbitrary points on structural planes can be acquired by using the oblique image technology of unmanned aerial vehicles. Then, the azimuth and distance of two arbitrary points can be solved based on the geographic coordinates. Combining with the corresponding elevation, the azimuth and apparent dip between the datum mark and a variety of points to be measured can be obtained. The maximum apparent dip and the corresponding azimuth can be selected as the attitude of a structural plane. Zhang [16] introduced the Gaussian mixture model–expectation maximization (GMM-EM) algorithm to calibrate parameters, including the trace length and dip angle of fractures. Song [17] designed the program for the mesh generation method of equatorial circles in the fractal dimension D of orientation pole distribution for joints. They also realized numerical representation of orientation pole maps for joints based on the conversion of polar coordinates at the orientation pole for joints with the dip direction and dip angle. This is conducive to the promotion and application of the fractal dimension D for orientation poles of joints. However, because smartphone image acquisition is limited in direct information acquisition of 3D structural planes, restoring 3D structural planes and determining joint attitudes according to known two-dimensional (2D) joint information are difficulties seldom studied in the previous intelligent identification process.

The research proposed a joint identification method on the surrounding rock surfaces of underground mine roadways based on the improved U-Net by summarizing the above research results. The PCP (three-point localization) algorithm was also used to rapidly calculate the dip and dip angle of joints on rock surfaces. Through application in an underground mine in China, the proposed identification technique of joint images and the attitude calculation method were verified to be feasible and accurate, and they can substitute artificial cataloging in the relatively simple underground environment.

The outstanding contributions of this paper are as follows:

(1) Smartphone images + RC-Unet enable pixel-accurate joint segmentation.
(2) CBAM + ASPP boosts thin-joint perception under uneven lighting.
(3) Image cutting + image rotation yields a large, balanced underground dataset.
(4) PCP converts 2D masks to 3D attitudes with ≈degree-level accuracy.
(5) Lightweight pipeline deploys easily in underground environments.

## 2. Joint Information Acquisition of Rocks Based on Smartphone Image Acquisition

### 2.1. Joint Information Acquisition Points of Rocks

**Figure 1:**
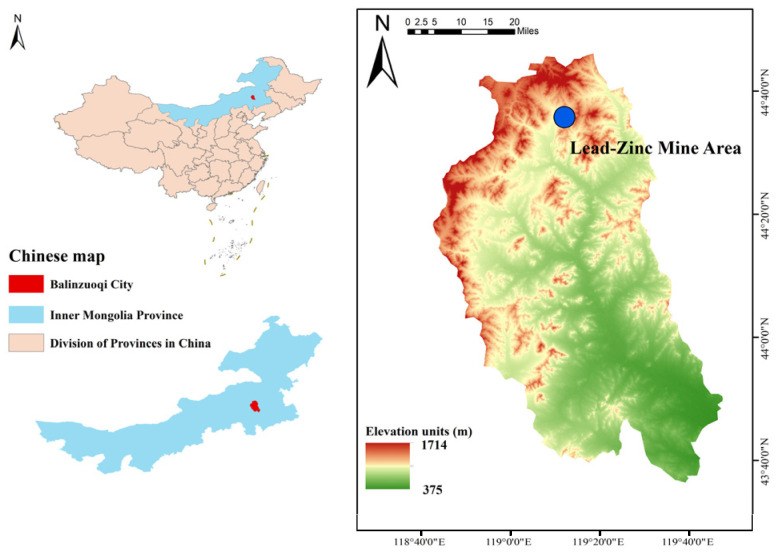
Sampling points.

**Figure 2:**
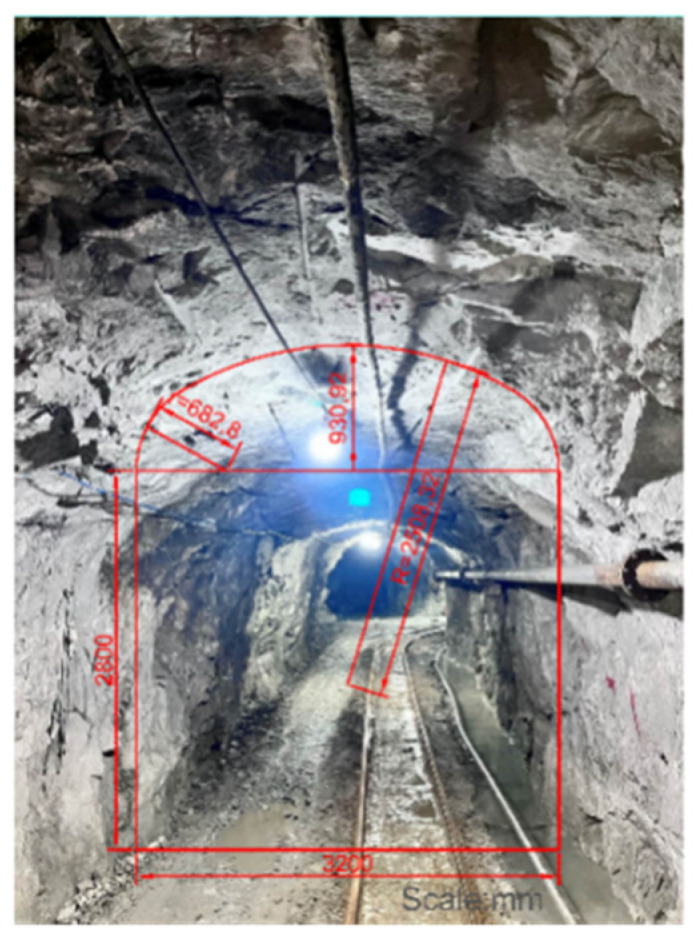
Front view of the roadway.

The research was based on a lead–zinc mine (119°13′09″~119°19′48″ E; 44°39′15″~44°36′32″ N) in Bairin Left Banner, Chifeng City, Inner Mongolia Autonomous Region, China. The mining area has a total length of 5.7 km, and the ore-bearing vein is 40 m~100 m wide, which is mainly distributed at the elevation of +500 m~+1100 m. The mineralized zone shows a strike of 55°~59° and dips to the north by west, with a steep dip angle (65°~85°). Photos of joints on the surrounding rocks of the underground roadway in the middle level of 705 m were mainly taken. The specific position is shown in Figure 1. The environment and dimensions of the roadway for taking photos are displayed in Figure 2. The roadway shows a three-center arch cross section, a width of 3.2 m, and a sidewall height of 2.8 m.

### 2.2. Data Acquisition

**Figure 3:**
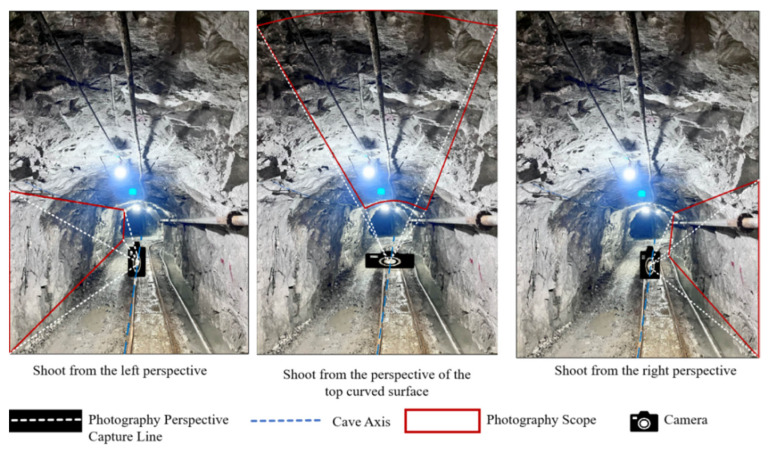
Sketch of information acquisition in the underground roadway of the mine.

A mobile phone was set up along the roadway axis. Areas with neat surrounding rock surfaces and obvious joint traces were selected for data acquisition. The total shooting length was about 3000 m. Data acquisition was completed by two researchers. One was responsible for photographing at each photography point along the roadway axis. In each area, photos of the same size were taken for three rock walls, namely, the left sidewall, top curved surface, and right sidewall. In this way, three 2D images of the 3D model in the area were obtained, as shown in Figure 3. When taking photos for the left and right sidewalls, it was necessary to ensure that the photographing height was the longitudinal elevation of 2.8 m of the roadway. When photographing the top curved surface, the photographing height should be the transverse elevation of 3.2 m of the roadway. The focal length could be changed according to the camera site during photographing, so as to achieve the desired shooting effect. There was a total of 60 groups, and namely, 180 photos were taken.

The other researcher was in charge of measuring and recording joints one by one in the area at each camera site using a geological compass and a diastimeter, including the strike, dip, dip angle, and length. The purpose was to calibrate rock information in the photos, and photos with unqualified sizes could be eliminated later. This could also provide data support for judging the accuracy of subsequent attitude calculation of joints.

**Figure 4:**
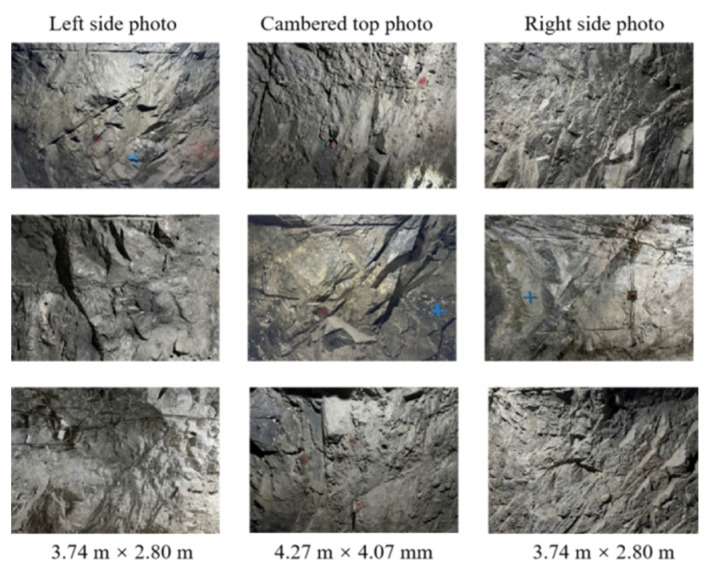
Partial samples obtained in data acquisition.

The lengths that can be determined in the photos include the following: the actual height of the left and right sidewalls is 2.8 m, and the width of the top curved surface is 3.2 m. Considering that the photos include 4096 × 3072 pixels (px), the aspect ratio is 4:3, so the actual size reflected by photos of the left and right sidewalls is 3.74 m × 2.80 m and that reflected by the projection line of the curved surface in photo of the top curved surface is 4.27 m × 3.20 m (4:3). According to the special surface proportional relation of three-center arches, the actual size reflected by photos of the top curved surface is 4.27 m × 4.07 m. Figure 4 shows examples of photos taken of the joints.

## 3. Dataset Creation

### 3.1. Preprocessing of Images in the Dataset

**Figure 5:**
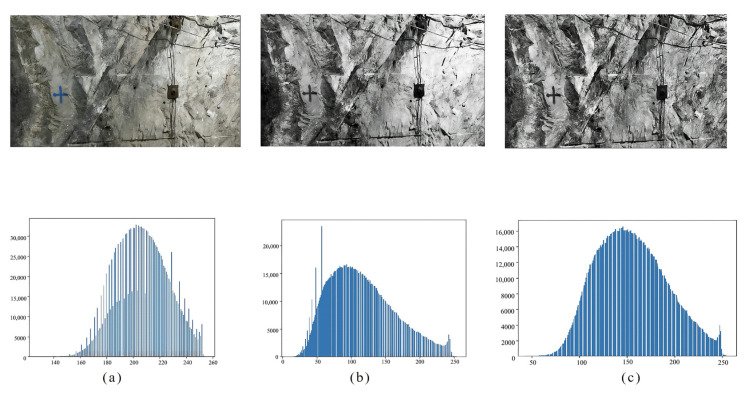
Comparison of the original photo with photos preprocessed using the two techniques. (**a**) Original photo; (**b**) photo processed using histogram equalization; (**c**) photo processed using CLAHE.

The images collected during mine production are generally of poor quality due to influences of factors including the complex mine environment, poor lighting conditions, angular deflection of the camera, and small scale of objects. Moreover, because it is a mobile phone that was used in the image, the images also show non-uniform gray distribution, low contrast, and unclear details, which directly influence the joint identification and analysis. Under these conditions, image enhancement techniques can be used to improve the image quality and enhance the joint identification accuracy and stability in images. Two image enhancement techniques, namely the histogram equalization and the contrast-limited adaptive histogram equalization (CLAHE) [18], are commonly used. Processing results of joint photos using the two methods are shown in Figure 5. By comparing the sharpness of photos processed using the two techniques, the research determined to use CLAHE as the preprocessing method of the rock wall photos.

Mine images typically suffer from non-uniform illumination, cast shadows, and highly textured backgrounds. Global histogram equalization (HE) often amplifies noise and washes out local contrast, whereas CLAHE enhances contrast locally while clipping histogram peaks to avoid noise amplification in near-uniform regions. As a result, edges of thin joints are better preserved, and background artifacts are less amplified. This behavior is well-established in the original CLAHE literature and subsequent applications. We therefore adopt CLAHE with a modest clip limit and tile grid (e.g., clipLimit ≈ 2–4, tileGridSize ≈ 8 × 8 in OpenCV) as a robust preprocessing step for underground images.

### 3.2. Image Data Augmentation

The drilling and blasting method was applied to underground tunneling, which left lots of corners on the surrounding rock surfaces. The boundaries of these corners are extremely easy to misjudge as joint contours. Therefore, to improve the accuracy of the model, the sliding window was used to cut photos, and photos with typical joint features were used to label the dataset. In the meantime, the image rotation method was used to augment the dataset in a bid to increase the sample size of the dataset.

#### 3.2.1. Data Augmentation by Image Cutting

Image cutting was utilized to process high-resolution joint photos in the dataset. It is assumed that the resolution of these high-resolution joint photos is *h* × *w* (px), where h and w separately represent the pixel values of the height and width of the photos. A fixed-size sliding window of *m* × *m* (px) was selected to cut an original photo into *n* × *a* × *b* low-resolution photos. After cutting, each photo has the same resolution of *m* × *m* (px). Invalid information of c (px) and d (px) along the height and width is separately cropped out from the high-resolution photo. The Equations are expressed as (1)–(4):

Suppose the original high-resolution image has a resolution of *h* × *w* (px). Given a fixed window size of *m* × *m* (px), the number of blocks along the height and width directions can be written as

(1) $$a=\left\lfloor \frac{h-c}{m}\right\rfloor ,\;\;b=\left\lfloor \frac{w-d}{m}\right\rfloor$$

where $\left\lfloor \cdot \right\rfloor$ denotes the floor operator.

The total number of cropped patches is *n* = *a* × *b*. The discarded pixels satisfy

(2) c=h mod m,  d=w mod m

where mod(*a*,*b*) denotes the remainder of integer division, taking values in the interval [0,*b*−1]. The row and column index ranges of the (*i*,*j*)-th block are

(3) rows: [im,im+m),  cols: [jm,jm+m),  0≤i<a, 0≤j<b

Accordingly, the mapping relationship is expressed as

(4) $$C(x,y)\mapsto T_{i,j}(x-im,\;y-jm),\;\;(x,y)\in [0,h)\times [0,w)$$

**Figure 6:**
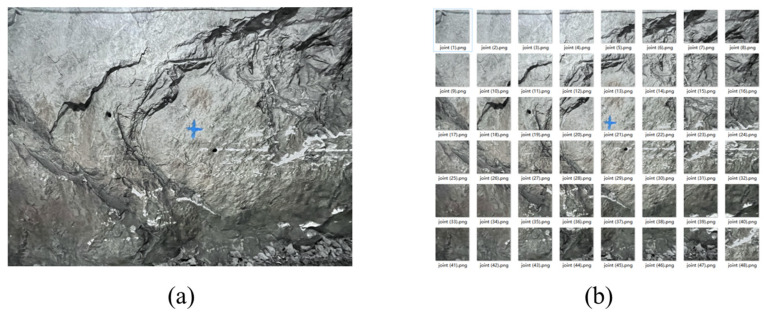
Sketches before and after using image cutting. (**a**) High-resolution joint photo taken in the field; (**b**) low-resolution joint images obtained using image cutting.

The original pixels of high-resolution joint photos taken in the research were 4096 × 3072 (px). A sliding window of 512 × 512 (px) was used. The pixel values c and d, cropped out, were ascertained according to the joint distribution in the photos. Figure 6a shows the actual photo taken; Figure 6b shows the image obtained using image cutting. After screening, finally, a total of 7200 images were obtained using image cutting.

#### 3.2.2. Data Augmentation by Using the Image Rotation Method

**Figure 7:**
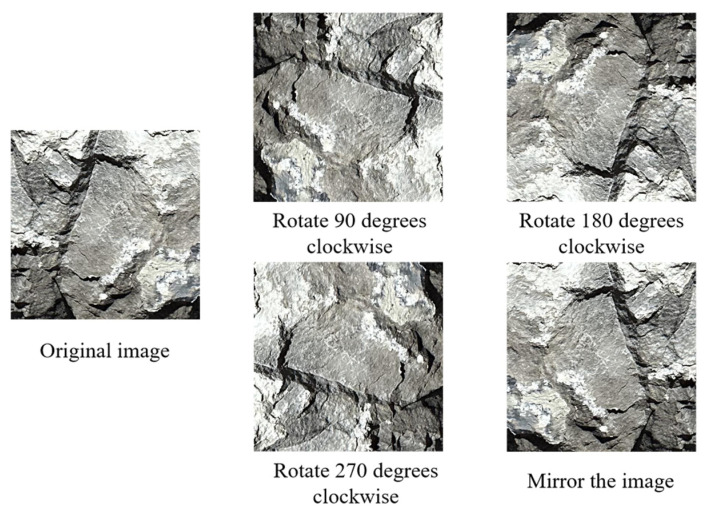
Sketch of the image rotation method.

The image rotation method was utilized for the expansion of small-sizesmall-sized joint images obtained using image cutting. Four copy images can be generated for each low-resolution joint image by clockwise rotating the image by 90°, 180°, and 270°, as well as flipping the image, as shown in Figure 7. By using this method, the number of images in the dataset can be expanded to five times that of the original image library. The method alters the location and trend of the joints. Each copy image is a new image of joint features that can be used subsequently to create the dataset.

### 3.3. Dataset Labeling and Partitioning 

**Figure 8:**
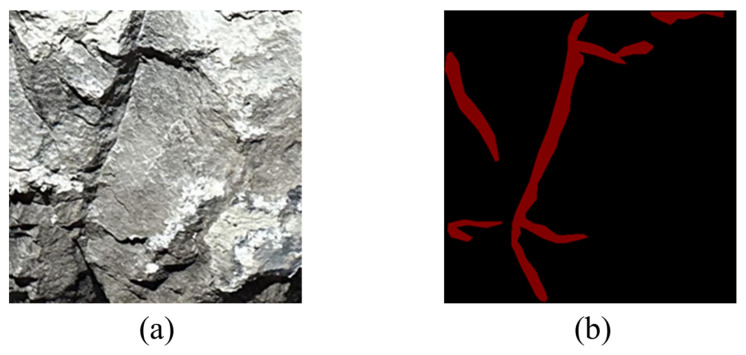
Sketch of joint labeling. (**a**) Original image; (**b**) labeled image.

Dataset creation is an operation of artificially labeling features in processed images, so as to obtain data for training the neural network. Labelme software(version 3.11.2) was adopted to manually delineate joints. In the process, joint images were magnified to be labeled point-by-point along joint contours. The area of the joints was labeled as a closed red polygon. After labeling, the database files were .json files, which could be converted into Portable-Network-Graphics-format label files that could be used to train the algorithm and model. Figure 8a,b separately display the joint image and the labeled image.

After applying image cutting to the high-resolution photos, 7200 images were obtained. After performing CLAHE in batch and using the image rotation method, the dataset can be expanded to 36,000 images. The joint and labeled images were divided into the training set, validation set, and test set with a ratio of 8:1:1. Finally, the number of images in the training set, validation set, and test set was separated into 28,800, 3600, and 3600, and the dataset was named Label-Joint.

## 4. Selection and Improvement of Joint Trace Identification Algorithms

### 4.1. Comparison and Selection of Algorithms

FCN, U-Net, and Seg-Net algorithms are effective semantic segmentation algorithms that are widely used at present. Each algorithm has specific advantages. FCN generally uses VGG16 as the encoder [19], while it does not consider the relationship between pixels. Especially when processing images of tiny and complex joints with much interference, the identification results of the algorithm exhibit low accuracy. Seg-Net is characterized by a decoding process that stores information of the maximum feature location in all pooling windows and provides a decoder for feature mapping [19]. It saves computer memory, improves performance, and shows a great advantage among lightweight neural networks. However, similar to FCN, the algorithm also does not consider the relationship between adjacent pixels and shows the disadvantage of sparsity, thus leading to inaccurate segmentation results. U-Net does not lose its boundary segmentation accuracy in the case of a small dataset in binary image segmentation tasks. The joint dataset studied in the research was acquired and created artificially, so the sample size and form of the dataset are also inadequate. U-Net can perfectly overcome the disadvantage of small datasets, whereas the model features a fixed structure, so it cannot be flexibly adjusted to adapt to joint identification and may also be overfitted. Considering this, it is necessary to improve the U-Net.

### 4.2. Improvement of the RC-Unet Model

The conventional U-Net structure is mainly composed of three parts: the compression path, the jump connection, and the expansion path [20]. The compression path includes the convolutional and pooling layers, which are responsible for size compression and feature extraction of input images. The expansion path consists of the deconvolutional and convolutional layers. It decodes features extracted in the compression path via the deconvolutional layer and restores the image size layer by layer. In the expansion path, a jump connection is used to splice the same network layers in the compression and expansion paths, so as to transfer more semantic information. The influences of multiple factors on the model performance need to be considered. These factors include the resolution of images input in the model, structural proportion of the model, size of convolution kernels, selection of the normalization method, types of loss and activation functions, and the potential introduction of the attention mechanism. By adjusting these parameters, the accuracy and generalization ability of the model can be effectively improved.

**Figure 9:**
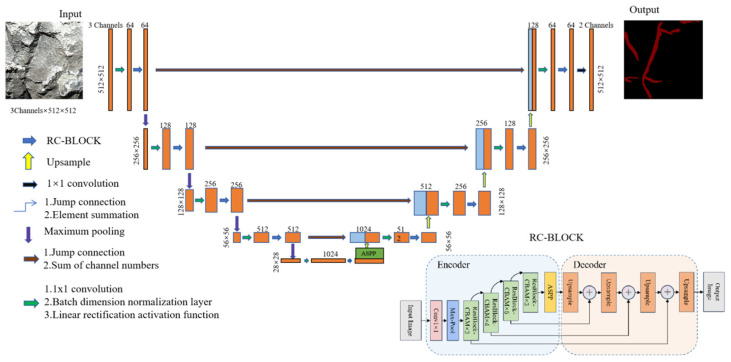
The improved RC-Unet model.

The improved RC-Unet model was proposed on the basis of the U-Net structure. The network model replaces the convolutional part with the RC-BLOCK formed by combining a residual connection module (Res-Net) and an attention mechanism module (CBAM). In addition, the ASPP module is added at the bottom of the model to further improve the perception ability of the model for joint features at different scales [20]. The overall structures are displayed in Figure 9.

The research then discussed the optimization strategies in detail below and verified their effects through experiments.

#### 4.2.1. Res-Net

Due to the complexity of joint features, U-Net is unable to completely extract joint features, limited by the depth of the network hierarchy. Appropriately increasing the depth of the network can enhance the joint identification ability of the model, while the conventional stacking of more convolutional layers can only result in the degradation of network performance. Res-Net retains a favorable feature acquisition capability while increasing the depth of the network [21]. The module increases the depth of the network by replacing the convolutional part in the compression path of U-Net with three 1 × 1, 3 × 3, and 1 × 1 convolutional layers, connected by a residual one. Residual connection enhances the interlayer connection and makes full use of the joint features in each layer. Experiments show that the structure can effectively capture the edge details and global structural features of joints and effectively solve the degradation of network performance caused by the increasing depth of the network.

#### 4.2.2. Introduction of the Attention Mechanism

CBAM, as a module of attention mechanism [22] that integrates the channel and space dimensions, adopts the following processing flow: the input feature map firstly generates the weighted feature map F_1_ via the channel attention sub-module, which is then input in the spatial attention sub-module to output the final weighted feature map F2, and the specific architecture is displayed in Figure 9. The research embeds CBAM in the residual module to enhance effective joint features while suppressing background noises (corners of rock blocks and construction marks), through the importance calibration in the channel dimension and selective focus on spatial areas. By adaptively adjusting the distributions of channel weight and spatial attention, the mechanism allows the model to precisely capture key structural features of joints in rocks, decrease interference of irrelevant information, and thus improve the feature representation ability and recognition accuracy in complex scenes.

**Figure 10:**
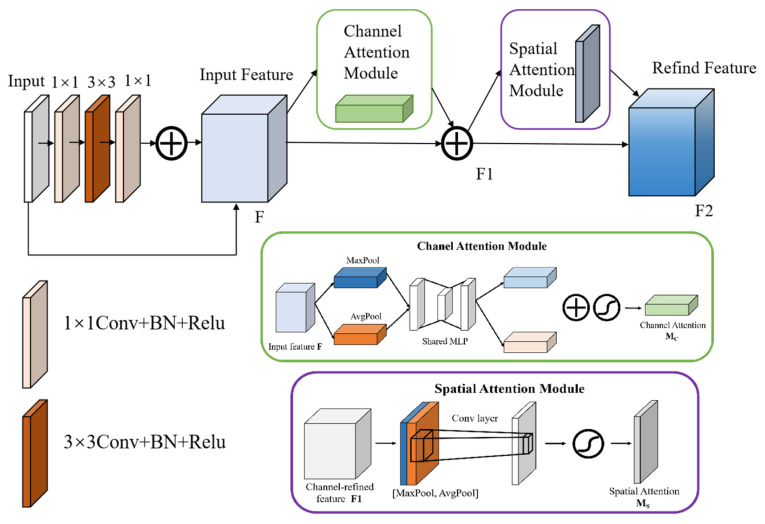
The Overall structure of the RC-BLOCK module.

The overall structure of the improved RC-BLOCK module is shown in Figure 10. The output features can be expressed as follows:

(5) $$F_{out}=F_{CBAM}\left(F_{in}+F_{A}\left(F_{in}\right)+F_{B}\left(F_{A}\left(F_{in}\right)\right)+F_{A}\left(F_{B}\left(F_{A}\left(F_{in}\right)\right)\right)\right)$$

where $F_{A}\left(•\right)$ is the convolution operation with a 1 × 1 convolution kernel; $F_{B}\left(•\right)$ is the convolution operation with a 3 × 3 convolution kernel; $F_{CBAM}$ refers to processing using the CBAM attention mechanism; and $F_{in}$ and $F_{out}$ separately represent the input and output features. Each convolutional layer is processed by a batch normalization (BN) layer and an activation function (ReLU). The BN and ReLU are conducive to alleviating vanishing gradient and overfitting caused by the increasing depth of the network.

#### 4.2.3. RC-Unet Workflow and Pseudocode

To clarify the internal mechanism of the proposed RC-Unet, we present the step-by-step forward pass together with a pseudocode description (Algorithm 1).
**Algorithm 1.** Forward Propagation of the Proposed RC-Unet$Input:I\in {\mathbb{R}}^{H\times W\times 3}$$1:X_{0}\leftarrow {\mathrm{Conv}}_{3\times 3}+\mathrm{BN}+\mathrm{ReLU}(I)$2:for l in {1..L}:$3:\;\;\;X_{l}\leftarrow \mathrm{RC}\_\mathrm{BLOCK}(\mathrm{Downsample}(X_{l-1}))$$4:Z\leftarrow \mathrm{ASPP}(X_{L})$5:for l in {1..L}:$6:\;\;\;Z\leftarrow \mathrm{UpSample}(Z)\;⊕\;\mathrm{Skip}(X_{l-1})$$7:\;\;\;Z\leftarrow {\mathrm{Conv}}_{3\times 3}+\mathrm{BN}+\mathrm{ReLU}(Z)$$8:P\leftarrow Sigmoid({\mathrm{Conv}}_{1\times 1}(Z))$Output:mask P

(6) $$Y=\mathrm{CBAM}({\mathrm{Conv}}_{1\times 1}({\mathrm{Conv}}_{3\times 3}({\mathrm{Conv}}_{1\times 1}(X))))+X$$

where CBAM applies channel attention followed by spatial attention. The ASPP module adopts parallel dilated convolutions with different rates to capture multi-scale context.

## 5. Effect Analysis and Indices of the Joint Identification Model

### 5.1. Parameter Selection

The computer hardware used in the experiment included the Intel® Core™ i7-10700 CPU, manufactured by Intel Corporation in Santa Clara, CA, USA, and the NVIDIA GeForce RTX 3070 GPU graphics card, produced by NVIDIA Corporation in Santa Clara, CA, USA, running on 64-bit Windows. As for the software, the experiment utilized TensorFlow-GPU 2.4.1, developed by Google LLC in Mountain View, CA, USA, was used, and the GPU was used for training. The resolution of images in the dataset was set to be 512 × 512 (px). Adam optimizer was employed to optimize the parameters, with the initial learning rate, batch size, and training batch separately set as 0.001, 2, and 100 iteration cycles. The validation set was used for verification after each iteration cycle, and the best training results were saved.

### 5.2. Selection of Loss Functions

The semantic segmentation of joint images is essentially a binary classification problem, in which the network must distinguish joint pixels (foreground) from background pixels. Due to the severe imbalance between the two categories—the joint regions are usually very sparse—using only the classical binary cross-entropy (BCE) loss often results in biased learning [23]. To alleviate this problem, we design a composite loss function that combines BCE and Dice losses, defined as

(7) $$L=\alpha L_{\mathrm{BCE}}+(1-\alpha )L_{\mathrm{Dice}},\;\;\alpha \in [0,1]$$

where α controls the balance between the two terms (default α = 0.5).

The BCE loss is given by

(8) $$L_{\mathrm{BCE}}=-\frac{1}{N}\sum_{n=1}^{N}[w_{1}y_{n}\log{\hat{y}}_{n}+w_{0}(1-y_{n})\log(1-{\hat{y}}_{n})]$$

where $y_{n}\in \{0,1\}$ is the ground-truth label of pixel *n*, ${\hat{y}}_{n}\in [0,1]$ is the predicted probability, and N is the number of pixels in the image. $w_{1}$ and $w_{0}$ are class weights introduced to reduce the effect of foreground–background imbalance.

The Dice loss is defined as

(9) $$L_{\mathrm{Dice}}=1-\frac{2\sum_{n=1}^{N}y_{n}{\hat{y}}_{n}+\epsilon }{\sum_{n=1}^{N}y_{n}+\sum_{n=1}^{N}{\hat{y}}_{n}+\epsilon }$$

where $\epsilon \in 10^{-6}$ is a small constant to ensure numerical stability.

In this formulation, BCE ensures probabilistic calibration and penalizes pixel-level misclassification but may suffer under severe class imbalance. Dice loss directly measures overlap quality between prediction and ground truth, which is particularly effective for capturing thin joint structures. By combining the two, the proposed composite loss balances stable optimization with accurate boundary segmentation, ensuring robust performance even when the joint pixels occupy only a very small proportion of the image.

### 5.3. Evaluation Indices

To quantitatively evaluate the segmentation effect of the model, the overall pixel accuracy $P_{A}$, intersection-over-union $I_{\mathrm{oU}}$, and $F_{1}\text{-}\mathrm{score}$ ($F_{1}$ value) were taken as test indices for model accuracy. The higher their values are, the better the segmentation effect. Therein, $I_{\mathrm{oU}}$ is the most referential index for assessing the segmentation effect of the model. $T_{P}$ and $F_{P}$ are the numbers of pixels in accurately or inaccurately predicted joint areas, and $T_{N}$ and $F_{N}$ are the numbers of pixels in the accurately or inaccurately predicted background, respectively.

$P_{A}$ and $I_{\mathrm{oU}}$ are separately expressed as follows:

(10) $$P_{A}=\frac{T_{P}+T_{N}}{F_{N}+F_{P}+T_{P}+T_{N}}$$

(11) $$I_{oU}=\frac{T_{P}}{F_{N}+F_{P}+T_{P}}$$

The $F_{1}$ value is the weighted average of the accuracy *P* and the recall rate *R* of the model, as expressed below:

(12) $$P=\frac{T_{P}}{F_{P}+T_{P}}$$

(13) $$R=\frac{T_{P}}{F_{N}+F_{P}}$$

(14) $$F_{1}=\frac{2PR}{P+R}$$

### 5.4. Model Comparison and Experimental Data Analysis

**Figure 11:**
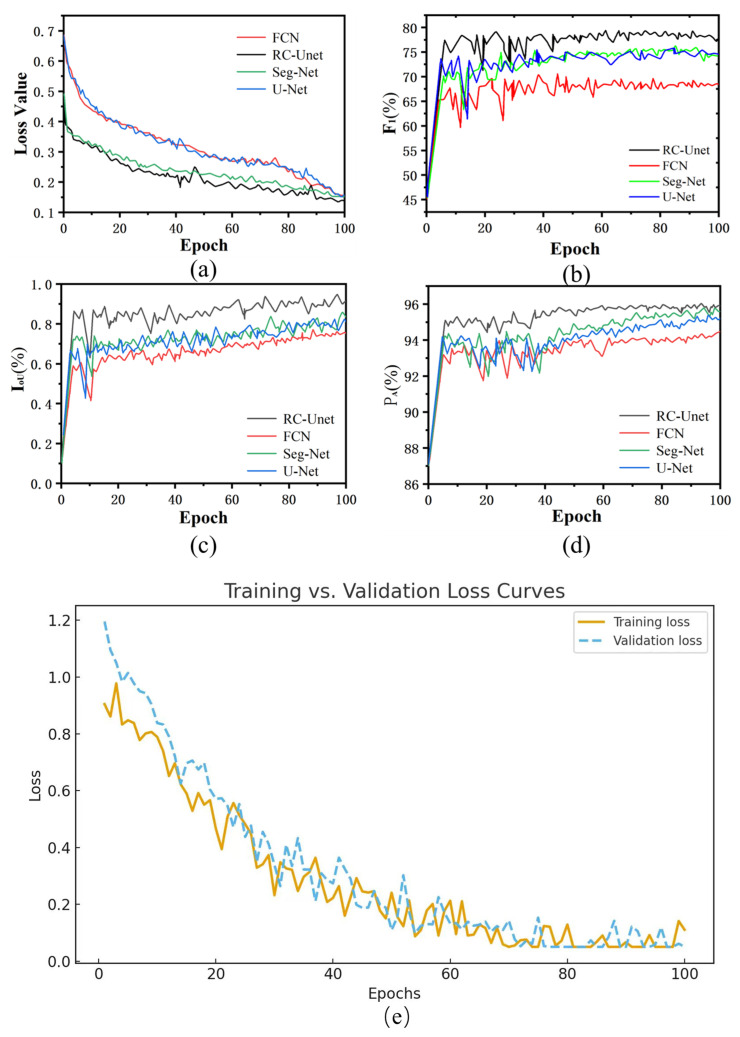
Change curves of various parameters. (**a**) Change curves of the loss function; (**b**) change curves of F1; (**c**) change curves of IoU; (**d**) change curves of PA; (**e**) comparison of training and validation loss curves over 100 epochs.

To verify the superiority of RC-Unet in joint segmentation, comparative experiments were conducted. The baseline semantic segmentation models U-Net, Seg-Net, and FCN, together with the improved RC-Unet, were trained and tested on the Label-Joint dataset. Their segmentation performances were then compared. After 100 training epochs, the changes in the loss function in the training set are illustrated in Figure 11a. As shown, the losses of all models gradually decrease and stabilize after about 90 epochs. Among them, RC-Unet achieves the lowest loss (0.147 at epoch 100), which is significantly lower than those of the other three models, demonstrating its superior convergence and fitting ability.

To further evaluate generalization, the variation curves of the F1-score, IoU, and PA on the validation set are presented in Figure 11b–d. In addition, Figure 11e compares the training and validation loss curves of RC-Unet. The validation loss follows a similar decreasing trend and converges consistently with the training loss, indicating that the model does not suffer from overfitting and maintains satisfactory performance on unseen data.

Changes in the indices within 100 iteration cycles were selected. RC-Unet, Seg-Net, U-Net, and FCN models differ in the number of iteration cycles for obtaining the optimal values of $F_{1}$, $I_{\mathrm{oU}}$, and $P_{A}$ indices. Optimal values of indices in the iteration process of various models are selected, as listed in Table 1. The highest values of $F_{1}$, $I_{\mathrm{oU}}$, and $P_{A}$ of the proposed RC-Unet model in the validation set are, separately, 78.97%, 94.55%, and 95.83%, which are all higher than the corresponding indices of the other three models.

### 5.5. Analysis of Classification and Identification Experimental Data of Joint Images

The images identified by the models were low-resolution images of 512 × 512 (px). Because it is high-resolution joint photos that were taken in the field, the photos need to be processed using the following method: one high-resolution joint photo needs to be cropped to multiple low-resolution joint images conforming to the identifiable size of the models via image cutting; then, RC-Unet is used to identify joints; finally, the segmented low-resolution joint images are respliced to a high-resolution overall joint image to calculate the attitude.

To analyze the identification accuracy of RC-Unet for different types of joints in low-resolution images and the superiority of RC-Unet to other semantic segmentation models, various images were classified into five different types based on the shape and density of joints. These five types were complex combined joints, simple combined joints, complex dense joints, simple dense joints, and simple sparse joints.

Table 2 compares the segmentation results of RC-Unet with Seg-Net, U-Net, and FCN models in the prediction set. Comparison of segmented images shows that in the case of a small number of joints with simple shapes, the four models can all accurately segment joints; once the joints have simple shapes with a large number, Seg-Net and FCN fail to accurately identify tiny joints. This is because of the small volumes and poor upsampling effects of Seg-Net and FCN, as well as the poor connectivity between pixels. U-Net yields relatively accurate identification results, and the identification accuracy differs slightly for the two types of joints. However, obviously, RC-Unet exhibits higher accuracy. In the case of joints with complex shapes, the identification results of FCN show a huge difference from reality, as evidenced by the large amount of detected leaks. The segments identified by Seg-Net and U-Net show large deviation, thus wrongly localizing many joints and being plagued with serious false detection. This is a result of the shallow spatial depth of the models and the lack of an attention mechanism. The proposed RC-Unet model increases the depth of the network by virtue of the residual module that fuses the CBAM attention mechanism, so it can better extract deep semantic features of joints. In this way, RC-Unet obtains more joint features and thus effectively avoids false and leak detection. The segmentation results of RC-Unet contain few noise interference points and show favorable continuity, with the width of the minimum identifiable joints being 2 pixels.

### 5.6. Ablation Experiments

Ablation experiments evaluate the necessity of each component for a model by analyzing changes in the performance after deleting a specific network module. The experiments combined U-Net (U) separately with the Res-Net module (R), ASPP module (A), and CBAM (C) to explore the influences of different module configurations on the semantic segmentation results, and the data are listed in Table 3.

Results show that IoU of RC-Unet is 10.58% higher than that of U-Net. IoU is improved when U-Net is separately fused with the RC or A module, while the model performance declines if the two modules are introduced simultaneously. This is because dilated convolution expands the receptive field, which enhances background interference, so that dense convolution fails to effectively extract joint features.

### 5.7. Statistical Validation

To further validate the superiority of the proposed RC-Unet, statistical significance testing was conducted on the per-image evaluation metrics. For each test image, F1-score, IoU, and PA values were calculated for RC-Unet, U-Net, Seg-Net, and FCN. The differences between RC-Unet and each baseline model were then examined using paired statistical procedures. Specifically, the normality of the paired differences was first assessed with the Shapiro–Wilk test. If the data followed a normal distribution, a paired t-test was applied; otherwise, the Wilcoxon signed-rank test was employed. To control for multiple comparisons across different metrics and models, the Holm–Bonferroni correction was used. Statistical significance was accepted at *p* < 0.05, and effect sizes (Cohen’s d) were also reported to assess the magnitude of improvement.

The results confirm that the performance gains of RC-Unet over U-Net, Seg-Net, and FCN are statistically significant. For example, in Table 1, RC-Unet achieves consistently higher values of F1, IoU, and PA, and the paired tests demonstrate that these improvements are not due to random variation (*p* < 0.01 for all three metrics). A similar conclusion is drawn from Table 3, where the ablation study shows that each added module contributes significantly to the overall performance of the framework. Effect size analysis further indicates medium-to-large improvements (Cohen’s d between 0.65 and 1.20), which confirms the practical relevance of the proposed method.

To provide a more detailed view, an additional table (Table 4) reports the mean differences, 95% confidence intervals, *p*-values, and effect sizes for the pairwise comparisons. These results demonstrate that the advantages of RC-Unet are robust and statistically supported.

### 5.8. Comparison with Recent Methods

Recent trends include foundation-/transformer-based segmenters and lightweight cracks/tunnel models [24,25]. For tunnel defects, YOLOv8-CBAM-style hybrids achieve strong multi-class detection/segmentation under good lighting yet rely on abundant labels and object-level priors. SAM/SAM-family models offer promptable masks but often lack robustness for hairline cracks in complex backgrounds without careful prompts or domain adaptation. Geological CT imagery has also seen DINOv2-based [26] transfer showing promise under scarce data. Compared with these, our RC-Unet focuses on pixel-accurate thin-structure extraction under non-uniform illumination typical of mines, leveraging CBAM for channel–spatial reweighting and ASPP for multi-scale context, with a 2D to 3D PCP pipeline for attitude recovery, which most generic segmentation works do not address.

## 6. Joint Data Extraction and Attitude Calculation

After reaching the stable identification effect using the RC-Unet model, it needs to extract 2D joint information on the surrounding rock surfaces of the roadway. Therefore, the Open-CV library was adopted for contour extraction of binarized joint images, so as to calibrate parameters, including the number of pixels and the minimum bounding oblique rectangle. Finally, these parameters were converted to actual geometrical parameters of joints, including the length, width, and dip angle.

The identified images are 4096 × 3072 (px) images with 12,582,912 pixels. The actual area represented by a unit pixel of images for the left and right sidewalls is 0.832 mm^2^, and that for the top curved surface is 1.381 mm^2^.

### 6.1. Statistical and Calculation Methods of Pixel Areas of Joints in the Area

The proportion of joints in an area reflects the degree of damage to surrounding rocks and is an important determinant index for quality grading of rocks [27]. Therefore, it is of extreme significance for calculating the proportion and area of joints.

The numbers of pixels of joints and other parts were counted, and the percentages of various parts in all pixels of an image were calculated. The specific operation is described as follows: two parameters with the initial value of 0 are constructed at first. They are labeled as black and red to separately represent the number of pixels other than joints and that of pixels in joint areas. Then, the for-loop is adopted to traverse images row by row and pixel by pixel. If a red pixel (within the upper and lower bounds of parameters in the HSV space) is detected, the value of red increases by 1; if a black pixel is detected, then the value of black increases by 1. The above steps are repeated until the detection is finished. The values of black and red are output, and the areal proportion of joints is automatically calculated. The calculation formula of the joint rate is shown as Equation (13):

(15) $$r=\frac{red}{black+red}\;$$

In this way, two general relations for calculating the areas of joints (or parts other than joints) in one image can be obtained, as shown in Equation (14). Then, the calculation formula for the area of joints is

(16) S=n×s 

where n is the number of pixels of joints (or parts other than joints) identified in images in the prediction set; s is the length of the actual range mapped by a single pixel in images in the prediction set.

Combining with the roadway size in the research, the calculation formulas for the area of joints in the whole area (unit: mm^2^) can be converted as follows:

(17) $$S_{side}=L_{side}\times W_{side}\times r_{side}$$

(18) $$S_{top}=L_{top}\times W_{top}\times r_{top}$$

Dimensions of the studied roadway are summarized as follows: L_side_, W_side_, Ltop, and W_top_ are, separately, 3740 mm, 2800 mm, 4270 mm, and 4070 mm; r_side_ and r_top_, separately, represent the fissure ratios of each side.

### 6.2. Statistical Method for the Length and Width of a Single Joint

Each of the 2D information of a single joint can be calculated as follows:

Edge recognition of joints (an edge refers to a polygon formed by connecting continuous pixels with the same gray scale using a line segment). The function can be realized through the library function Find-Contours, and then the library function Contour-Area is used to calculate the area of the contours. The specific implementation method is described as follows: the Green formula is applied to binarized images to calculate the areas according to the area enclosed by edges.

**Figure 12:**
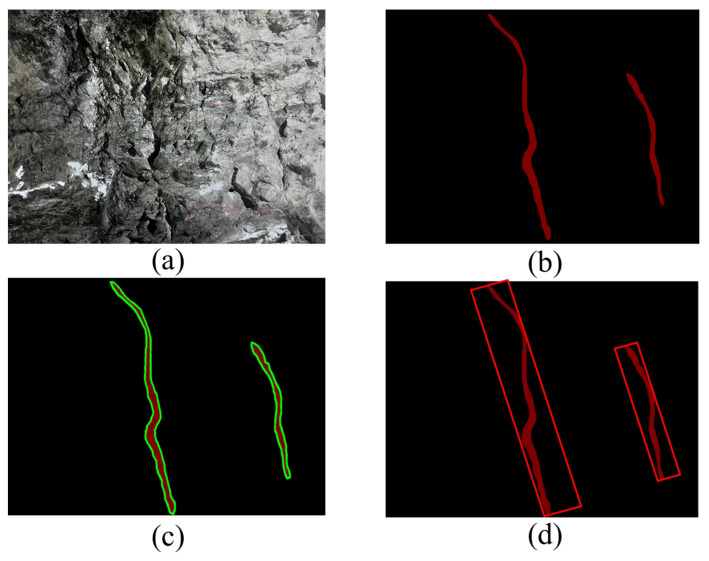
Effect images after processing using various algorithms. (**a**) Original image; (**b**) binarized image identified by RC-Unet; (**c**) contour identification using the Find-Counters algorithm; (**d**) determining the minimum bounding rectangle using the Min-AreaRect algorithm.

Function Min-AreaRect is adopted to obtain the minimum bounding rectangle that completely covers the contours. Figure 12 shows the process to determine the minimum bounding rectangle. Function Box-Points is used to attain the height, width, and rotation angle (θ), as well as four vertices of the rectangle.

**Figure 13:**
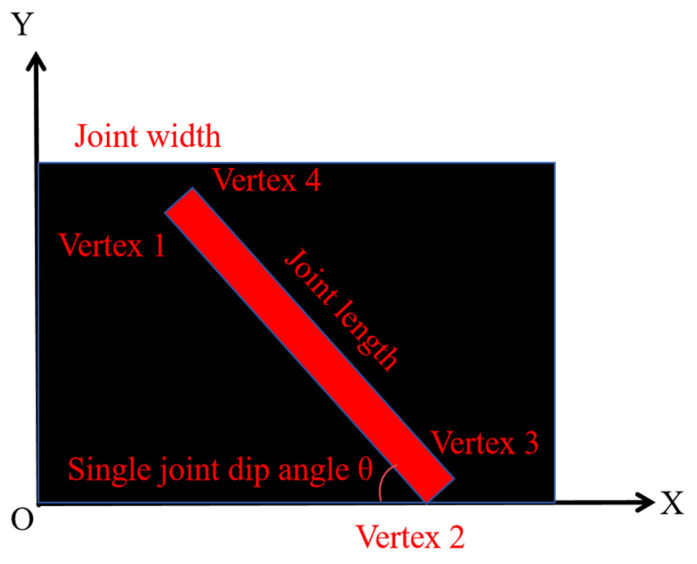
Acquisition of geometrical parameters of joints.

After acquiring the contours and basic parameters of the minimum rectangle, a rectangular plane coordinate system is established at the lower left corner of the image in the actual size. In this way, the coordinates of four vertices of the bounding rectangle on joints on the left sidewall, right sidewall, and top curved surface, namely, (xLij, yLij), (xRij, yLij), and (xTij, yTij) (i refers to the ith joint in the image; j is the jth point on the ith joint), are attained. Then, the length li, width di, and dip angle ai (angle $\theta_{i}$ between the long side and the x-axis) of a single joint are acquired through coordinate calculation, as shown in Figure 13.

### 6.3. Attitude Calculation of 3D Joint Planes Using the PCP Algorithm

**Figure 14:**
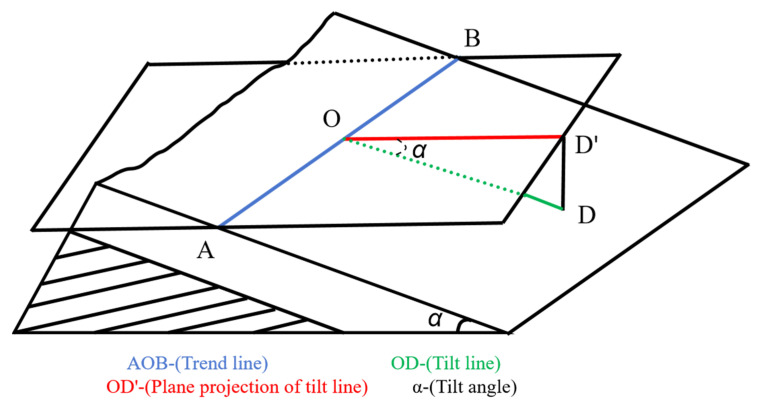
Essentials for the attitude of rocks.

The attitude of rocks is determined by the spatial extension direction and the inclination of a rock plane, and it is represented by values of essentials, including the strike, dip, and dip angle of the rock plane (Figure 14).

To determine the attitude of rocks, it is necessary to ascertain the plane on which the rocks are localized. Obviously, a rock plane can be determined and the attitude can be measured after obtaining the coordinates of three (or more) points on the rock plane [28,29].

Therefore, the PCP algorithm was used for rapid localization of joint planes and parametric characterization of the attitude. For each joint on the plane, the central point of the minimum bounding rectangle is used to replace the position of the joint on the plane. Then, it matches with a joint on the adjacent plane. A 3D joint plane is formed by connecting the central substitution points of three joints. This plane is the single joint plane, of which the attitude needs to be calculated. Each joint plane in the area is determined by permutating and combining each joint on each rock wall in the area. The formulas for determining the plane coordinates of the three points are as follows:

The substitution point for joints on the left sidewall is

(19) $$\left(x_{Li},y_{Li}\right)=\left(\frac{x_{Li1}+x_{Li2}+x_{Li3}+x_{Li4}}{4},\frac{y_{Li1}+y_{Li2}+y_{Li3}+y_{Li4}}{4}\right)$$

The substitution point for joints on the right sidewall is

(20) $$\left(x_{Ri},y_{Ri}\right)=\left(\frac{x_{Ri1}+x_{Ri2}+x_{Ri3}+x_{Ri4}}{4},\frac{y_{Ri1}+y_{Ri2}+y_{Ri3}+y_{Ri4}}{4}\right)$$

The substitution point for joints on the top curved surface is

(21) $$\left(x_{Ti},y_{Ti}\right)=\left(\frac{x_{Ti1}+x_{Ti2}+x_{Ti3}+x_{Ti4}}{4},\frac{y_{Ti1}+y_{Ti2}+y_{Ti3}+y_{Ti4}}{4}\right)$$

Here, $\left(x_{i}^{L},y_{i}^{L}),\;(x_{i}^{R},y_{i}^{R}),\;(x_{i}^{T},y_{i}^{T}\right)$ denote the center coordinates (in pixels) of the minimum bounding rectangle of joint i in the left, right, and top images, respectively.

**Figure 15:**
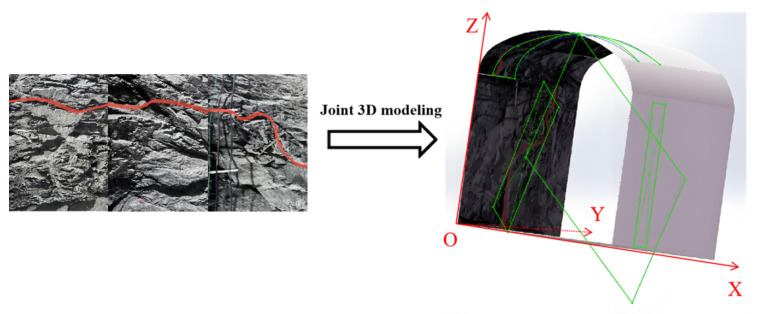
Sketch of 3D joint planes.

After acquiring substitution points of joints on each rock wall, it also needs to obtain parameters of substitution points in the spatial coordinate system by transforming the plane-local spatial coordinates. As shown in Figure 15, a 3D coordinate system is established on the roadway floor at the lower left corner. XYZ is the 3D spatial coordinate system in the photographing area, and xyz is the local 2D plane coordinate system of the photos taken. Spatial parameters of joint planes in the 3D spatial coordinate system are calculated using the following formulas:

Spatial coordinates of substitution points on the left sidewall are

(22) $${\left[X_{Li},Y_{Li},Z_{Li}\right]}^{T}={\left[0,x_{Li},y_{Li}\right]}^{T}$$

Spatial coordinates of substitution points on the right sidewall are

(23) $${\left[X_{Ri},Y_{Ri},Z_{Ri}\right]}^{T}={\left[l,x_{Ri},y_{Ri}\right]}^{T}$$

where l is the cross sectional length of the roadway, which is 3200 mm in the research.

Spatial coordinates of substitution points on the top curved surface are

(24) $${\left[X_{Ti},Y_{Ti},Z_{Ti}\right]}^{T}={\left[3200-0.786y_{Ti},x_{Ri},\phi \left(y_{Ti}\right)\right]}^{T}\;\;{\left[X_{Ti},Y_{Ti},Z_{Ti}\right]}^{T}={\left[l-\varepsilon y_{Ti},x_{Ri},\phi \left(y_{Ti}\right)\right]}^{T}$$

where ε is the proportionality coefficient of the roadway (0.786 here), which is substituted to obtain

(25) $${\left[X_{Ti},Y_{Ti},Z_{Ti}\right]}^{T}={\left[3200-0.786y_{Ti},x_{Ri},\phi \left(y_{Ti}\right)\right]}^{T}$$

where $\omega \left(y_{Ti}\right)$ and $\phi \left(y_{Ti}\right)$ are composite functions containing $\phi \left(y_{Ti}\right)$. $\phi \left(y_{Ti}\right)$ and $\lambda \left(y_{Ti},\;\phi \left(y_{Ti}\right)\right)=0$ are met in the studied roadway, in which $\phi \left(y_{Ti}\right)$ is calculated as follows:

(26) $$\lambda \left(y_{Ti},\;\phi \left(y_{Ti}\right)\right)={\left(\omega \left(y_{Ti}\right)-P_{i}\right)}^{2}+{\left(\omega \left(y_{Ti}\right)-P_{k}\right)}^{2}-{R_{i}}^{2}=0$$

After substituting the research data, the calculation formula is expressed as Equation (27):

(27) $$\lambda \left(y_{Ti},\;\phi \left(y_{Ti}\right)\right)=\left\{\begin{array}{l} {\left(2512-0.786y_{Ti}\right)}^{2}+{\left(\phi \left(y_{Ti}\right)-2800\right)}^{2}-682.8^{2}=0\;\;\;0\leq y_{Ti}<340\\ {\left(1600-0.786y_{Ti}\right)}^{2}+{\left(\phi \left(y_{Ti}\right)-1221\right)}^{2}-2508.32^{2}=0\;340\leq y_{Ti}\leq 2860\\ {\left(688-0.786y_{Ti}\right)}^{2}+{\left(\phi \left(y_{Ti}\right)-2800\right)}^{2}-682.8^{2}=0\;\;2860<y_{Ti}\leq 3200 \end{array}\right.$$

The midpoints of three joints in the area are connected to form a straight joint plane. In the roadway area, the roadway floor at the lower left corner is selected to construct the 3D coordinate system, thus obtaining coordinates of three points, namely, $M_{Li}\left(X_{Li},Y_{Li},Z_{Li}\right)$, $M_{Ri}\left(X_{Ri},Y_{Ri},Z_{Ri}\right)$, and $M_{\mathrm{Ti}}\left(Z_{Ti},Z_{Ti},Z_{Ti}\right)$. Based on the coordinates of the three points, the equation of the joint plane can be solved as Equation (28):

(28) $$AX_{i}+BY_{i}+CZ_{i}+D=0$$

Then, the dihedral angle $\alpha_{i}$ between the joint plane and the plane of the roadway floor is calculated. At first, the normal vectors $\vec{n_{i}}={\left(n_{i1},n_{i2},n_{i3}\right)}^{T}$ and $\vec{n_{j}}={\left(0,0,1\right)}^{T}$, separate from the joint plane and the roadway floor, are calculated:

(29) $$\vec{M_{Li}M_{Ri}}\cdot \vec{n_{i}}=\left(X_{Ri}-X_{Li},Y_{Ri}-Y_{Li},Z_{Ri}-Z_{Li}\right)\times {\left(n_{i1},n_{i2},n_{i3}\right)}^{T}=0$$

(30) $$\vec{M_{Li}M_{Ti}}\cdot \vec{n_{i}}=\left(X_{\mathrm{Ti}}-X_{Li},Y_{Ti}-Y_{Li},Z_{Ti}-Z_{Li}\right)\times {\left(n_{i1},n_{i2},n_{i3}\right)}^{T}=0$$

According to the formula for the dihedral angle $\alpha_{i}$,

(31) $$\alpha_{i}=\arccos\frac{\vec{n_{i}}\cdot \vec{n_{j}}}{\left|\vec{n_{i}}\right|\times \left|\vec{n_{j}}\right|}$$

The azimuth representation is used. The dip angles $\alpha_{m}$ of the plane of the roadway floor and the azimuths $\beta_{m}$ (m is the number of planes of roadway floors with different data) for representing the dip in each construction stage are input in advance. Therein, the final actual attitude of the joint plane is represented as $\beta_{m}∠\alpha_{n}$, in which $\alpha_{n}$ is calculated using the following formula:

(32) $$\alpha_{n}=\left\{\begin{array}{l} \alpha_{i}+\alpha_{m}\;\;\;\;0\leq \alpha_{i}+\alpha_{m}\leq \frac{\pi }{2}\\ \left|\pi -\left(\alpha_{i}+\alpha_{m}\right)\right|\;\;\;\alpha_{i}+\alpha_{m}>\frac{\pi }{2} \end{array}\right.$$

By using this method, the attitudes of multiple joint planes in the photographing area of the roadway can be localized.

### 6.4. Analysis of Joint Attitude Calculation Results

After obtaining the attitude of joint planes through batch computing, attitude data of rock faces with 50 joints measured using the 3D joint scanner in the field were selected and compared with those calculated using the algorithm. Considering the length of the table, Table 5 shows 10 groups of data selected from 50 groups of joint surface data.

(33) $$\bar{\left|\Delta \alpha \right|}=\frac{1}{M}\sum_{i=1}^{M}|{\hat{\alpha }}_{i}-\alpha_{i}|,\;\;\bar{\left|\Delta \beta \right|}=\frac{1}{M}\sum_{i=1}^{M}|{\hat{\beta }}_{i}-\beta_{i}|$$

After 50 groups of verification, it is calculated that the average error $\bar{\Delta \beta }$ of azimuth for representing the dip and the average error $\bar{\Delta \alpha }$ of the dip angle are

$$\bar{\Delta \beta }=\frac{1}{50}\sum_{m=1}^{50}\left({\beta_{m}}^{\prime }-\beta_{m}\right)=\frac{-103.8}{50}=-2.076{}^{\circ}$$

$$\bar{\Delta \alpha }=\frac{1}{50}\sum_{m=1}^{50}\left({\alpha_{n}}^{\prime }-\alpha_{n}\right)=\frac{97.2}{50}=1.944{}^{\circ}$$

**Figure 16:**
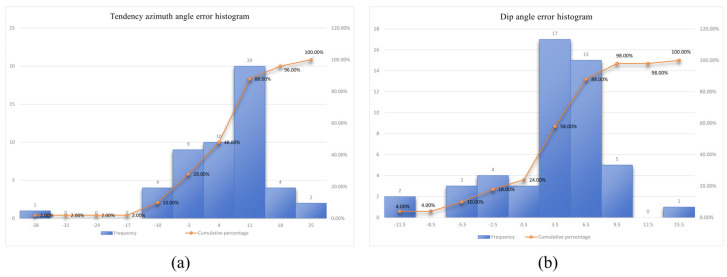
Histograms for the two groups of data. (**a**) Histogram for the error of the azimuth for representing the dip; (**b**) histogram for the error of the dip angle.

Figure 16a,b displays histograms for the average error of azimuth for representing the dip and the average error of dip angle.

Calculation results show that the average calculation error of the attitude of the 50 joint planes is relatively low (2°). According to the histogram distribution, the attitude errors of more than 80% of joint planes are within an extremely small range of 10°, meeting the requirement for excavation faces in the mine production standard. This proves that the algorithm basically conforms to the actual attitude of joint planes in the practical production process and can be applied to practical production.

## 7. Conclusions

The research achieved intelligent identification of joints in the underground rock mass based on a lead–zinc mine in Bairin Left Banner, Chifeng City, Inner Mongolia Autonomous Region, China. The following conclusions are obtained:

(1) To solve problems of artificial geological cataloging, including the low efficiency and susceptibility to subjective factors, the established multi-module RC-Unet was used for intelligent identification of joints in underground rock mass. Comparison with artificially drawn results reveals that the accuracy of intelligent identification is higher than 90%, so it can be used as an auxiliary means of geological cataloging.
(2) RC-Unet shows a low loss in joint identification, which is only 0.147. In addition, F1, IoU, and PA indices are all superior to those of FCN, Seg-Net, and U-Net models. Therefore, RC-Unet exhibits more obvious applicability to joint identification.
(3) The PCP attitude algorithm based on the Open-CV library yields results that agree well with artificial measurements. Comparison shows that the errors of 50 joints tested are lower than 2°, which means that the PCP attitude algorithm is applicable to the geological description of the underground surrounding rocks.
(4) With the rapid development of artificial intelligence and smartphone hardware, the artificial geological cataloging in the complex underground environment can be gradually replaced. The research findings can not only reduce the operational risks for underground workers, but also provide a new idea for the big-data collection of geological information and intelligent auxiliary analysis. They are of great significance for the intelligent construction of mines.

Limitations and Practical Considerations

(1) The accuracy of 3D attitude calculation is bounded by the 2D segmentation quality; joints narrower than ~2 px or severely occluded may be missed.
(2) CLAHE parameters and exposure vary across sites; domain shifts caused by camera devices, lighting, or lithology require light re-tuning or fine-tuning.
(3) The PCP plane assumption presumes locally planar walls and a valid arch proportion (λ); strong curvature or camera pose errors may degrade 3D mapping accuracy.
(4) Compared with SAM-/transformer-based approaches, RC-Unet is lighter and more deployment-friendly underground, but it lacks promptable interaction and large-scale pretraining. Future work will explore SAM-/DINOv2-style adapters for low-shot adaptation and self-calibration using multi-view constraints.

## Figures and Tables

**Table 1:** Comparison of evaluation parameters of the four algorithms.

Model	F1 (%)	IoU (%)	PA (%)
FCN	70.56	76.18	94.03
U-Net	75.18	83.97	95.38
Seg-Net	76.78	85.16	95.67
RC-Unet	78.97	94.55	95.83

**Table 2:** Comparison of image recognition effects of the four algorithms.

Sequence	Image	Label file	RC-Unet	U-Net	Seg-Net	FCN
Complex combined joints	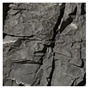	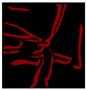	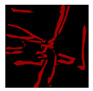	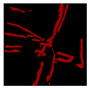	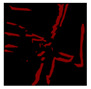	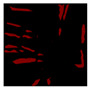
Simple combined joints	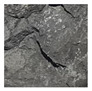	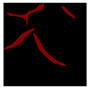	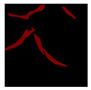	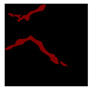	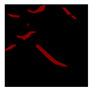	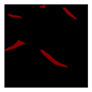
Complex dense joints	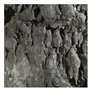	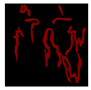	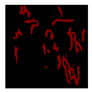	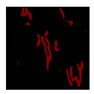	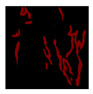	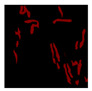
Simple dense joints	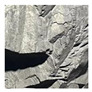	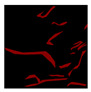	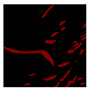	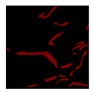	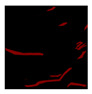	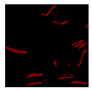
Simple sparse joints	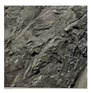	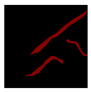	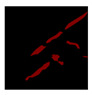	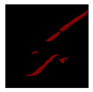	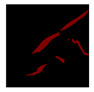	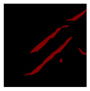

**Table 3:** Segmentation results of different models.

Combination	U	U + A	U + RC	U + RC + A
IoU (%)	83.97	84.12	94.55	77.22

**Table 4:** Statistical validation of RC-Unet performance against baseline models.

Metric	Comparison	Mean Difference	95% CI	*p*-Value	Effect Size (d)
F1	RC-Unet vs. U-Net	+0.07	[0.04,0.10]	0.001	0.85
IoU	RC-Unet vs. Seg-Net	+0.05	[0.03,0.08]	0.002	0.70
PA	RC-Unet vs. FCN	+0.06	[0.02,0.09]	0.004	0.65

**Table 5:** Comparison between 3D scanner measurements and algorithmic calculations for 10 representative joint planes (azimuth and dip; units: °).

seq	Scanner Dip (°)	Scanner Azimuth (°)	Algorithm Dip (°)	${\alpha_{n}}^{\prime }$ (°) Algorithm Azimuth (°)	$\Delta \beta ={\beta_{m}}^{\prime }-\beta_{m}$ (°)	$\Delta \alpha ={\alpha_{n}}^{\prime }-\alpha_{n}$ (°)
1	156.5	76.9	157.3	77.4	0.8	0.5
2	186.7	15.3	189.5	16.5	2.8	1.2
3	351.2	8.9	359.3	17.4	8.1	8.5
4	125.2	19.6	136.3	25.6	11.1	6.0
5	268.4	58.6	274.1	74.1	5.7	15.5
6	114.2	23.6	118.7	15.2	4.5	−8.4
7	214.6	44.4	223.6	49.6	9.0	5.2
8	341.2	59.6	336.9	47.3	−4.3	−12.3
9	187.6	6.3	196.3	14.7	8.7	8.4
10	25.3	29.9	30.2	35.6	4.9	5.7
⋯⋯						

## Data Availability

The source code can be download from GitHub repository (https://github.com/zhujinyao0724/Joint_Identification_Model/blob/main/Joint_identification_Model.rar (accessed on 4 October 2025)) only for research purpose.
